# Precursor N-cadherin mediates glial cell line-derived neurotrophic factor-promoted human malignant glioma

**DOI:** 10.18632/oncotarget.15302

**Published:** 2017-02-12

**Authors:** Ye Xiong, Liyun Liu, Shuang Zhu, Baole Zhang, Yuxia Qin, Ruiqin Yao, Hao Zhou, Dian Shuai Gao

**Affiliations:** ^1^ Department of Neurobiology and Anatomy, Xuzhou Key Laboratory of Neurobiology, Jiangsu Key Laboratory of New Drug Research and Clinical Pharmacy, Xuzhou Medical University, Xuzhou 221004, Jiangsu, China; ^2^ Key Laboratory of Industrial Ecology and Environmental Engineering (Ministry of Education), School of Food and Environmental, Dalian University of Technology, Panjin Campus, Panjin 124221, China

**Keywords:** precursor N-cadherin, glioma cells, GDNF, migration, invasion

## Abstract

As the most prevalent primary brain tumor, gliomas are highly metastatic, invasive and are characteristic of high levels of glial cell-line derived neurotrophic factor (GDNF). GDNF is an important factor for invasive glioma cell growth; however, the underlying mechanism involved is unclear. In this study, we affirm a significantly higher expression of the precursor of N-cadherin (proN-cadherin) in most gliomas compared with normal brain tissues. Our findings reveal that GDNF interacts with the extracellular domain of proN-cadherin, which suggests that proN-cadherin mediates GDNF-induced glioma cell migration and invasion. We hypothesize that proN-cadherin might cause homotypic adhesion loss within neighboring cells and at the same time promote heterotypic adhesion within the extracellular matrix (ECM) through a certain mechanism. This study also demonstrates that the interaction between GDNF and proN-cadherin activates specific intracellular signaling pathways; furthermore, GDNF promoted the secretion of matrix metalloproteinase-9 (MMP-9), which degrades the ECM via proN-cadherin. To reach the future goal of developing novel therapies of glioma, this study, reveals a unique mechanism of glioma cell migration and invasion.

## INTRODUCTION

The metastatic and invasive growth characteristics of malignant glioma are the main reasons for the disease's poor prognosis. The biological processes of tumors migration and invasion are the results of the interaction between tumor cells and their microenvironment, which is regulated by many factors, such as various trophic factors. Glial cell-line derived neurotrophic factor (GDNF), a member of the transforming growth factor-β (TGF-β) superfamily, is a soluble extracellular factor initially found to be a protective factor for the survival and differentiation of dopaminergic neurons [1]. It was later found to be involved in the development of gliomas. GDNF levels are significantly higher in human brain malignant glioma than normal human brain tissue fluid [2–3]. Many studies have confirmed that GDNF also plays important roles in the metastatic and invasive processes of the glioma. It was reported to significantly improve Hs683 glioma cell migration and invasion [4]. Researchers have linked nutritional factors to the development of human glioma [2, 5–6]. GDNF performs its functions primarily via the Ret/GDNF family receptor α1 (GFRα1) receptor complex [7–8]. Our previous study preliminarily demonstrated that integrin β1 and neural cadherin (N-cadherin) adhesion molecules on the cell membrane can also act as the signal transduction receptors of GDNF to activate PI3K/AKT signaling [9–10]. Although the correlation between GDNF and glioma has received various attentions, the interaction between GDNF and N-cadherin requires deeper exploration.

N-cadherin is a homophilic transmembrane adhesion glycoprotein that is widely distributed in the central nervous system neurons and glial cells. Its intracellular domain can be linked to the actin cytoskeleton by different catenin molecules; moreover, it can also act as a transmembrane signal transduction receptor to transmit signals that influence intercellular adhesion [11]. In our previous researches, we reported that in the dopaminergic nerve cell line MN9D, the phosphorylation level of N-cadherin (Tyr860) correlated with GDNF concentration in a time-dependent manner and that N-cadherin can mediate the effects of GDNF on intracellular signaling pathways [10].

The invasive growth of tumor cells depends on decreased adhesion between individual tumor cells, and also greater adhesion between tumor cells and the ECM. However, the questions are, why is the homophilic binding ability of N-cadherin on tumor cell membranes reduced while the heterophilic binding ability is enhanced, and there is no such process in the normal cell membrane? Are there structural changes in N-cadherin?

N-cadherin expression in glioma is significantly increased compared to normal mammalian brain tissue [12, 13]. ProN-cadherin is the precursor form of the mature protein. The former has 159 amino acids (AAs) more than the latter (746AA) [14], and both can co-exist on the same cell membrane [15]. Using different World Health Organization (WHO)-grade glioma cell lines, western blot analysis shows a high proportion of proN-cadherin at the cell surface of high-grade U251 glioma cell line compared to low-grade U343 glioma cell line [15], however, there is no record of proN-cadherin protein expression in solid glioma tissues or other glioma cell lines.

Based on the existing data, we hypothesize that a considerable portion of N-cadherin in glioma cell membranes reported in papers published before the year 2010 is actually proN-cadherin since authors might not have distinguished between proN-cadherin and N-cadherin. It is increased proN-cadherin expression that reduces intercellular adhesion and increases adhesion between cells and the matrix. In this study, we demonstrate that proN-cadherin mediates GDNF-promoted malignant astroglioma cells migration and clarify a novel molecular mechanism underlying glioma cell invasion and migration, which might influence future clinical diagnosis and treatments for glioma.

## RESULTS

### ProN-cadherin is abundant in the cytomembrane of most gliomas

**Figure 1:**
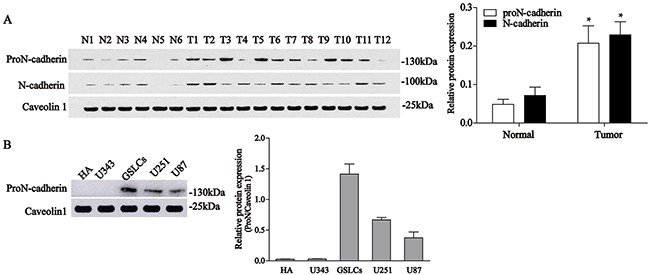
Expression levels of proN-cadherin and N-cadherin in normal brain, glioma tissues, and 5 cell lines **A**. Western blot analyses of proN-cadherin and N-cadherin expression in 12 glioma samples (T1-12) and 6 normal brain samples (N1-6). Caveolin 1 was used as internal control. Values were compared with unpaired t-tests. *P<0.05. **B**. Protein expression levels of proN-cadherin in the cytomembrane of 5 cell lines.

To examine the expression differences between proN-cadherin and mature N-cadherin in different glial cells and tissues, we selected two antibodies with different epitopes: the antigen epitopes of the antibodies were the extracellular domain of mature N-cadherin and the pro region of proN-cadherin. We then performed western blot analysis to explore the expression profiles of proN-cadherin. The experimental results showed that 9 out of 12 glioma tumor samples display higher proN-cadherin amounts than in the control samples, whereas the remaining 3 tumor samples express lower proN-cadherin (Figure 1A, Supplementary Table 1), this is consistent with results of the microarray analysis that N-cadherin mRNA levels are higher in the majority of GBMs than normal human brain [13]. We also compared proN-cadherin expression in 5 different cell lines: human astrocyte (HA), U343, U251, U87, and glioblastoma stem-like cells (GSLCs). The results showed that proN-cadherin is highly expressed in the U251, U87 and the GSLCs, with the highest expression recorded in the cytomembrane of the latter compared with HA and U343 cell lines (Figure 1B).

### GDNF promotes U251 malignant glioma cell migration and invasion

**Figure 2:**
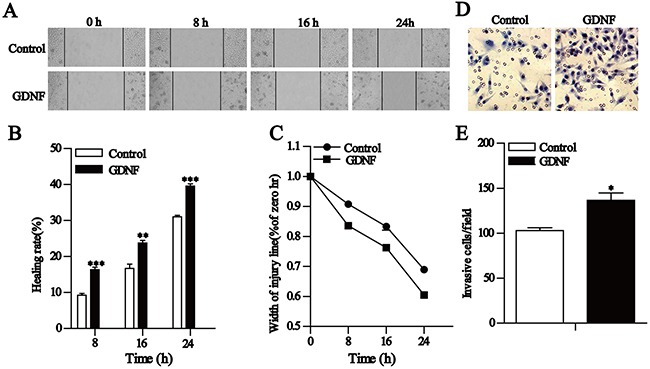
GDNF promotes U251 cell migration and invasion *in vitro* **A-C**. Wound healing assays show that GDNF promotes U251 cell migration. A Representative images (×200) taken at different time points. B Cell motility was quantified by measuring the wound width. The degree of motility is presented as a percent of migration at the zero time point. C. Changes in U251 cell healing rates at different time points. **D** and **E**. Transwell assays of U251 cells showing the effect of exogenous GDNF. D Representative fields (×200) of invasive and migratory cells on the membrane. E Quantitative analysis of the invasive and migratory cells from three independent experiments. Error bars represent SEM. *P<0.05, **P<0.01, ***P<0.001

GDNF is not only a trophic factor but also a chemoattractant for various cells [16–20]. More importantly, it is highly expressed in human gliomas [2–3]. To extend our findings we performed wound healing and transwell invasion assays to determine whether exogenous GDNF affects the migration and invasion of U251 malignant glioma cells. Wound healing assay results showed that the healing rates of the 50 ng/ml GDNF group at 8, 16, and 24 h after scraping significantly increased compared to that of the control group (Figure 2A, 2B), and the relative width of the scraping decreased in a time-dependent manner (Figure 2C). Our transwell invasion experiment results revealed that the number of penetrated cells after treatment with GDNF for 24 h significantly increased compared with the control group (Figure 2D, 2E). Consistently, exogenous GDNF significantly promoted U251 malignant glioma cell migration and invasion.

### GDNF and proN-cadherin could interact with each other

**Figure 3:**
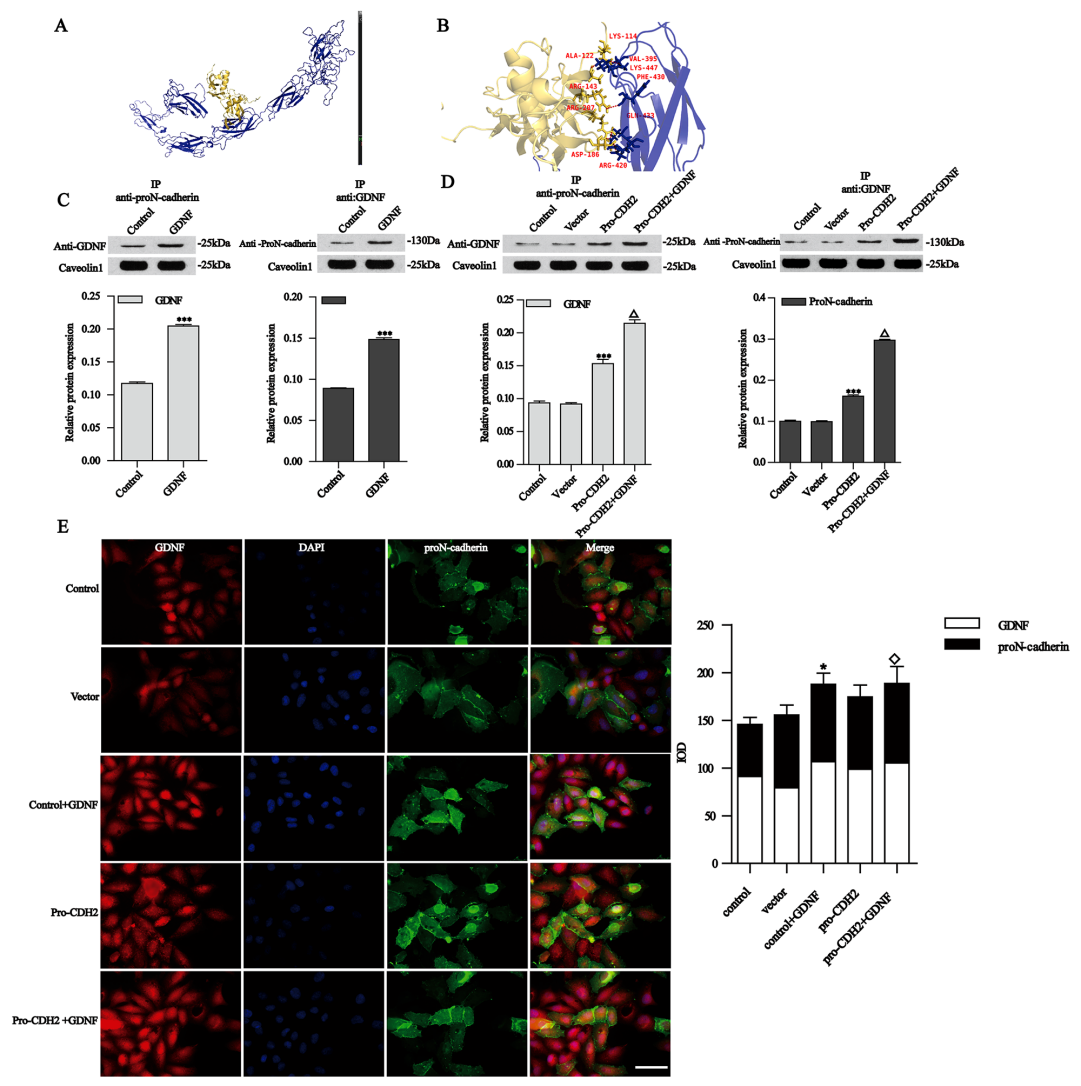
Simulation diagram for the interaction between GDNF and proN-cadherin **A**. Fully rendered diagram. **B**. Zoomed image showing the multiple amplification effects. Blue indicates proN-cadherin, yellow indicates GDNF, and five pairs of hydrogen bond are shown (Lys114-Val395, Ala122-Lys447, Arg143-Phe430, Arg207-Gln433, and Asp186-Arg420). **C** and **D**. Co-immunoprecipitation and western blotting analysis of GDNF and proN-cadherin. Caveolin 1 was used as an internal control. **E**. Immunofluorescence images and analysis of the proN-cadherin and GDNF interaction. Immunofluorescence experiments showing GDNF (red, ×400), proN-cadherin (green, ×400), and merged images of control U251 cells, cells transfected with vector or pro-CDH2, and U251 cells treated with exogenous 50 ng/ml GDNF for 30 min. DAPI (blue, ×400) was used to stain nuclei. ProN-cadherin localized in the cell membrane and cytoplasm, while GDNF was in the cytoplasm. Bar, 50 μm. Data of the relative expression of proN-cadherin and GDNF in different groups of U251 cells are presented as mean ±SEM. *P < 0.05 compared with the control group, ◇ *P* < 0.05 compared with the vector group.

To determine whether the precursor protein proN-cadherin can transmit GDNF signals into the cell, we simulated the interaction between these two proteins using protein-protein docking software (hex8.0.0 software), we found that the binding energy was -337 kcal/mol, and GDNF could form hydrogen bonds with five AA residues on the EC3 of proN-cadherin (the hydrogen bond figure was prepared using PyMOL [www.pymol.org], and the distance between hydrogen donors and acceptors was determined to be less than 3.2 Å) (Figure 3B). The primary docking results of GDNF and proN-cadherin showed that they could interact with each other (Figure 3A, 3B).

Our data indicated that proN-cadherin was highly expressed at the cell surface of malignant astroglioma. Since proN-cadherin lacks adhesion properties [21], we assumed that the loss of cell adhesion might be due to abnormally high expression of proN-cadherin, which may lead to cell motility and allow GDNF to promote U251 cells migration. In order to explore how proN-cadherin affected malignant astroglioma cells’ migration, U251 malignant glioma cell models with different proN-cadherin concentrations in the cytomembrane were established to carry out a series of experiments. Quantitative polymerase chain reaction (Q-PCR) and western blot analysis showed that proN-cadherin over-expression and silencing were successful in U251 cells (Supplementary Figure 1).

Then we verified the interaction between the two molecules by co-immunoprecipitation (Co-IP). The results showed that proN-cadherin interacted with GDNF (Figure 3C, control vs control). Furthermore, the GDNF and proN-cadherin contents in groups treated with 50 ng/ml GDNF for 30 min were higher than those in control group (Figure 3C, GDNF vs control, P<0.001 respectively), indicating that increased GDNF concentration significantly promoted its interaction with proN-cadherin. We demonstrated that GDNF and proN-cadherin could co-exist. Based on this understanding, we explored how the contents of proN-cadherin changed, and how this affected its interaction with GDNF by transfecting the proN-cadherin plasmid into U251 cells, then we performed western blots and immunoprecipitation assays respectively. Western blot results showed higher GDNF and proN-cadherin protein levels compared with the control group (Figure 3D, *pro-CDH2* vs vector, P<0.001). U251 cells transfected with proN-cadherin plasmid were then treated with 50 ng/ml GDNF for 30 min followed by Co-IP. The Co-IP analysis showed that GDNF and proN-cadherin protein levels were higher in the transfected/GDNF-treated group compared with the control groups (Figure 3D, *pro-CDH2*+GDNF vs *pro-CDH2*, P<0.001, respectively), indicating that both over-expression of proN-cadherin and exogenous GDNF treatment enhanced the interaction between GDNF and proN-cadherin, further supporting our hypothesis on GDNF and proN-cadherin interaction.

We later performed immunofluorescence (IF) experiments to localize protein expression within cells and further verify the interactions between proN-cadherin and GDNF. The experimental results showed they could interact with each other in the cytoplasm, and this interaction was significantly enhanced after treatment with 50 ng/ml GDNF for 30 min (Figure 3E, GDNF vs control, P<0.05). The fluorescence intensities of GDNF and proN-cadherin were slightly enhanced after transfection with a proN-cadherin plasmid (Figure 3E, *pro-CDH2* vs vector, *P*>0.05). Moreover, combining these two together (transfection of proN-cadherin plasmid and 50 ng/ml exogenous GDNF treatment) for 30 min enhanced this interaction compared with the vector group (Figure 3E, *pro-CDH2*+GDNF vs vector, P<0.05). These experiments reveal that GDNF and proN-cadherin could interact with each other.

### GDNF promotes U251 malignant glioma cell migration mediated by proN-cadherin directly

**Figure 4:**
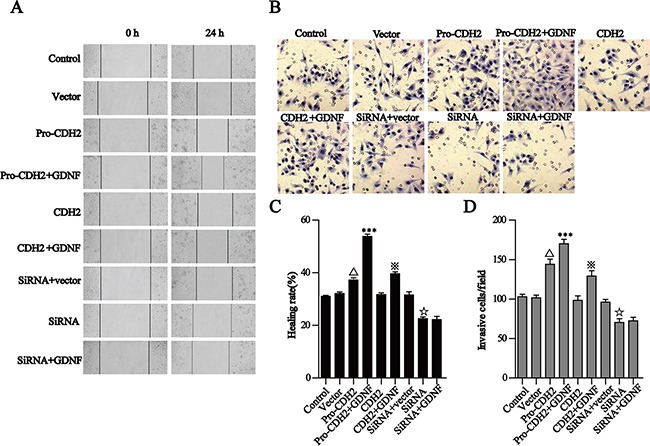
GDNF promotes U251 cell migration and invasion via proN-cadherin *in vitro* **A, C**. The wound healing assay showed that GDNF promotes U251 cell migration through proN-cadherin. A Representative images (×200) were taken at different time points. C Quantification of cell motility by measuring wound width. The amount of motility was presented as a percent of migration at the zero time point. **B** and **D**. Transwell assays of U251 cells transfected different vectors. B Representative fields (×200) of invasive and migratory cells on the membrane. D Quantitative analysis of the invasive and migratory cells from three independent experiments. Error bars represent the SEM. ^△^p<0.001 compared with the vector, ***P<0.001 compared with the pro-CDH2 group, ^※^ P<0.001 compared with the CDH2 group,^☆^P<0.01 compared with the siRNA +vector group.

Based on our confirmation that proN-cadherin interacts with GDNF, we speculated that proN-cadherin acts as a functional transmembrane signaling molecule to directly transmit extracellular GDNF signals into cells, thus promoting cell invasion and migration. To test this hypothesis, we carried out wound healing assays and transwell invasion experiments. The results of the former showed that 24 h after scraping, the healing rate in the proN-cadherin over-expression group (*pro-CDH2*) was significantly higher than that of the vector control group, unlike the overexpression of N-cadherin which had no influence (P>0.05). Furthermore, the healing rate in the proN-cadherin pro-segment silencing group (siRNA-*pro-CDH2*) was obviously reduced (30%). Although exogenous GDNF promoted healing in the *pro-CDH2* and CDH2 over-expression groups, the healing rate in the *pro-CDH2*+GDNF group (41%) was significantly increased compared with that of the *CDH2*+GDNF group (19%). Interestingly, GDNF treatment did not affect the healing rate in the proN-cadherin pro-segment silencing group (siRNA-*pro-CDH2*+GDNF). These results suggest that the presence or absence of the pro-segment of proN-cadherin affected healing during GDNF-induced glioma cell migration (Figure 4A, 4C).

Similar to the results of wound healing assay, the transwell experiment results showed significantly increased number of penetrating cells at 24 h in the proN-cadherin over-expression group compared with the vector group, indicating that proN-cadherin increment independently promoted cell invasion. Also, the number of penetrating cells in the *pro-CDH2*+GDNF group was greater than that of the pro-CDH2 group. Although the number of penetrating cells significantly decreased after silencing the pro-segment of proN-cadherin (siRNA-*pro-CDH2*), there was no difference following GDNF treatment (+GDNF), while N-cadherin overexpression did not significantly affect cell invasion as compared with the vector. Collectively, these data support the hypothesis that GDNF promotes U251 cell migration and indicate that proN-cadherin plays important roles as a functional molecule (Figure 4B, 4D).

### Enhanced heterophilic adhesion between U251 glioma cells and ECM

During brain glioma invasion, adhesion between tumor cells and ECM induces the secretion of matrix-degrading enzymes, resulting in local degradation of normal brain tissue and promoting local invasion [22]. N-cadherin levels are important in the invasion and migration of glioma. The stable overexpression of N-cadherin full-length cDNA alters the adhesion properties of C6 cells with ECM, especially type I collagen [13].

**Figure 5:**
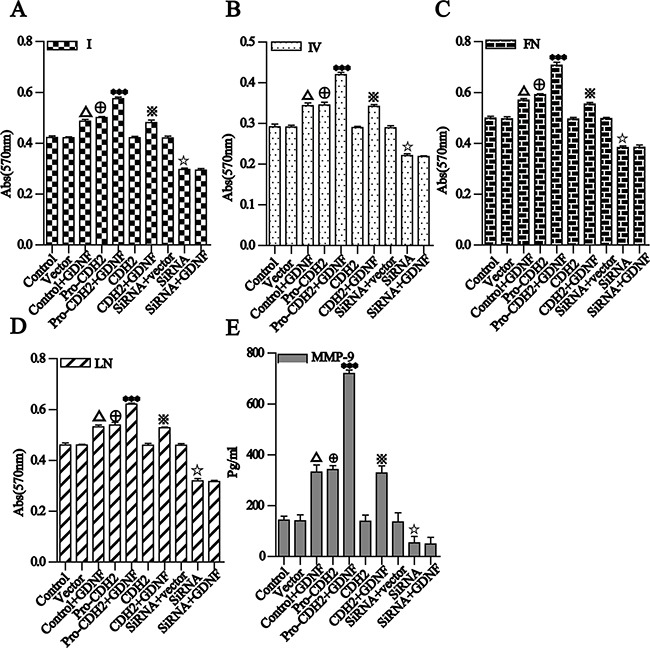
Different groups of U251 cell adhesion to four kinds of ECM proteins and detection and analysis of MMP-9 secretion **A** and **B**. for I, IV collagen, **C** and **D**. for fibronectin and laminin. Different groups of U251 cells were placed in 96-well plates pre-coated with the same concentrations of the four ECMs. After a 2-h incubation and removal of unattached cells, the adherent cells in each well were determined by the absorbance as described in the methods. **E**. MMP-9 levels in the culture supernatants were assayed by ELISA. The data are expressed as mean ± SD. P<0.001 compared with the control group, P<0.001 compared with the vector group, ***P<0.001 compared with the pro-CDH2 group, P<0.001 compared with the CDH2 group, P<0.001 compared with the siRNA+vector group.

To detect changes in the adhesion between cells and ECM during proN-cadherin-GDNF mediated cell invasion and migration, we examined adhesion between U251 cell groups with four ECM proteins (collagen I and IV, laminin, and fibronectin). We observed that adhesions among the cells in each group were significantly enhanced 30 min after GDNF treatment. Notably, when proN-cadherin contents of cytomembrane are different, adhesion between the cell and the four ECM proteins showed a positive linear relationship to cell membrane content of proN-cadherin. Over-expression of proN-cadherin and increased GDNF content synergistically and significantly increased cell adhesion; however, N-cadherin overexpression had no effect. These results show that proN-cadherin is involved in promoting adhesion properties of U251 cells, and GDNF also promoted U251 cell adhesion. Both effects were cumulative, and changes in N-cadherin content did not affect the cell adhesion (Figure 5A-5D).

### U251 glioma cells secrete more MMP-9

MMPs belong to an important proteolytic enzyme family that degrades ECM and basement membrane components as well as some non-matrix components to alter the microenvironment of tumor cells and enhance their invasion and metastasis [23–24]. We detected MMP-9 secretion by the U251 cell groups which have different proN-cadherin expressed in their cytomembranes with an MMP-9 ELISAs Kit. The results showed that after the changes of proN-cadherin contents in the cell surface by over-expression or silencing, the amount of MMP-9 secreted by U251 cells showed a positive linear relationship with proN-cadherin content in the cell membrane (*pro-CDH2 vs*. vector, siRNA-*pro-CDH2 vs*. siRNA vector, both P<0.001). Conversely, N-cadherin overexpression did not affect MMP-9 secretion (*CDH2 vs*. vector, P>0.05). The cells over-expressing proN-cadherin and N-cadherin were treated with GDNF, and MMP-9 expression nearly doubled in the former and was not significantly changed in the latter. MMP-9 secretion was significantly reduced by proN-cadherin silencing (siRNA-*pro-N-CDH2 vs*. siRNA vector), which was not restored by GDNF treatment. These results suggest that GDNF promotes MMP-9 secretion by U251 cells, which is mediated by proN-cadherin (Figure 5E).

### Specific signaling pathways participate in proN-cadherin-mediated, GDNF-promoted glioma cell migration

**Figure 6:**
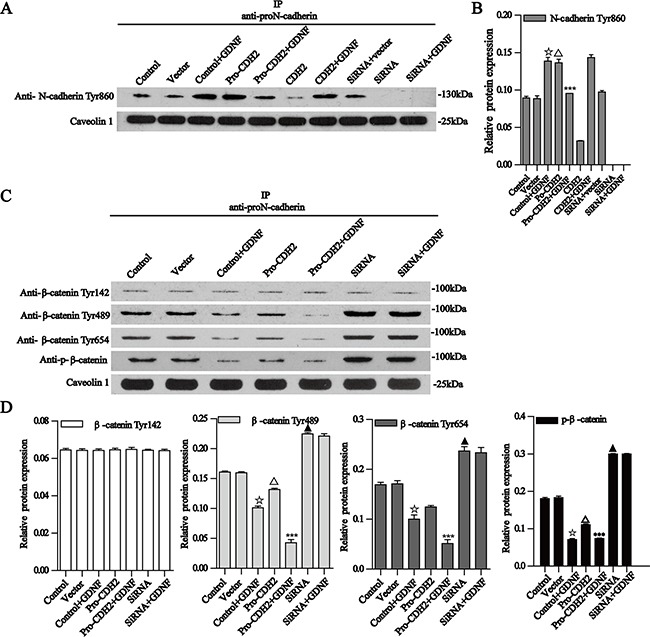
Co-immunoprecipitation of proN-cadherin with N-cadherin Tyr860 and β-catenin Tyr142, Tyr489, Tyr654, and p-β-catenin **A**. Co-immunoprecipitation of proN-cadherin with N-cadherin Tyr860. ProN-cadherin antibodies for immunoprecipitation, N-cadherin Tyr860, antibody for western blot analysis. Caveolin 1 was used as an internal control. **C-D** Bar graphs show the relative protein expressions. Data are presented as the mean±SD for three independent experiments. **B**. Co-immunoprecipitation of proN-cadherin with β-catenin Tyr142, Tyr489, Tyr654, and p-β-catenin Ser33/37/Thr41. ProN-cadherin antibodies for immunoprecipitation, β-catenin Tyr142, Tyr489, Tyr654, and Ser33/37/Thr41antibodies for western blot analysis. Caveolin 1 was used as an internal control. B-E Bar graphs show the relative protein expressions. Data are presented as mean± SEM for three independent experiments. ☆P<0.001 compared with the control group, Δ P<0.001 compared with the vector group, ***P<0.001 compared with the pro-CDH2 group, ▲ P<0.001 compared with the control group.

We also explored the intracellular GDNF signaling pathways mediated by proN-cadherin. To determine whether the intracellular domain of proN-cadherin had changed, we examined the phosphorylation status of Tyr860 in the intracellular domain of proN-cadherin with western blot analysis after immunoprecipitation using an antibody whose epitope belongs to the pro region of proN-cadherin and found significant differences among different U251 groups. The phosphorylation level of Tyr860 in proN-cadherin was increased in GDNF-treated U251 cells; it is also increased after proN-cadherin plasmid transfection. Collectively, these results suggest that Tyr860 phosphorylation in the intracellular domain of proN-cadherin is positively regulated by the interaction between GDNF and proN-cadherin. However, in over-expressed proN-cadherin U251 cells with GDNF-treatment, the phosphorylation level of this site barely changed compared with the blank vector group, indicating that there might be a threshold on the phosphorylation level of Tyr860 in proN-cadherin. That is, Tyr860 phosphorylation would not be further increased if the strength of the interaction between proN-cadherin and GDNF exceeded a certain level. In addition, after transfection with proN-cadherin siRNA, Tyr860 phosphorylation in proN-cadherin was not detected even after GDNF treatment. This result further suggests that Tyr860 phosphorylation in the intracellular region of proN-cadherin is positively regulated by the pro region of proN-cadherin that interacts with GDNF (Figure 6A, 6B).

As β-catenin and p120-catenin interact with the cytoplasmic sequences of N-cadherin, we carried out western blot after immunoprecipitation of proN-cadherin and further found that GDNF did not significantly change the binding capacities between the intracellular proN-cadherin domain and the above two molecules (data not shown). Hyperphosphorylation of β-catenin has consistently correlated with loss of adhesive function [25], so we extracted U251 cell membrane proteins and examined four β-catenin phosphorylation sites (*i.e*., Tyr142, Tyr489, Tyr654, and Ser33/37/Thr41). We found out that the phosphorylation level of Tyr142 was not significantly changed by the interaction between proN-cadherin and GDNF, but phosphorylation of the Tyr489, Tyr654, and Ser33/37/Thr41 sites was obviously decreased when proN-cadherin is over-expressed or exogenous GDNF treated (Figure 6C, 6D).

After GDNF treatment, the phosphorylations of Tyr489, Tyr654, and Ser33/37/Thr41 sites in β-catenin were reduced, and a similar tendency was observed after proN-cadherin plasmid transfection. Moreover, the phosphorylations of the three tyrosine sites were significantly decreased by the combination of proN-cadherin plasmid transfection and GDNF treatment. These results suggested that the increased interaction between GDNF and proN-cadherin reduced phosphorylation of Tyr489, Tyr654, and Ser33/37/Thr41 sites in β-catenin. In the GDNF-treated group, over-expressed proN-cadherin group, and over-expressed proN-cadherin + GDNF-treated groups, the phosphorylation levels of Tyr489 and Tyr654 sites in β-catenin were significantly reduced when the interaction between GDNF and proN-cadherin was increased, which was in agreement with a previous study. That is, the phosphorylation of Tyr654 in β-catenin was negatively related to the integrity of the cadherin-catenin complex [26], suggesting that the interaction between cadherin and β-catenin was stronger when GDNF levels were higher and the interaction between GDNF and proN-cadherin was strengthened (Figure 6C, 6D).

## DISCUSSION

Our analyses of membrane proteins revealed that proN-cadherin was higher in 9 out of 12 glioma tissue samples compared with normal brain tissues, which indicates that proN-cadherin is abundant in the majority of glioma tissues. The remaining 25% of glioma tissues which showed lower expression of proN-cadherin might be low-grade glioma or non-astroglioma cells. Further experiments verified that malignant astroglioma cell lines (U87 and U251) as well as GSLCs have high expression of proN-cadherin while no similar phenomenon was detected in neither low-grade glioma cell line U343 nor a normal human astrocyte. Since researchers didn't distinguish proN-cadherin from the total N-cadherin in the majority of past documents, we suggest the data of N-cadherin contents in different glial cell surfaces could to some extent mirror a trend of proN-cadherin expression. Two different researchers reported that N-cadherin expression in glioma was significantly higher than normal mammalian brain tissues [12, 13], which is consistent with our western blot results of N-cadherin and hints the credibility of our western blot analysis of proN-cadherin. However, there was an inverse correlation between the expression of N-cadherin and the pejorative WHO-grade of the glioma in surgical specimens and the N-cadherin-positive rate of recurrent glioma was lower compared to that of primary glioma [12, 13]. This might explain why proN-cadherin is not highly expressed in all glioma cell surfaces. More different grades of human glioma tissues samples should be provided to detect their proN-cadherin expressions to explore this protein in the future.

Signatures of genomic abnormalities can define different tissue subgroups, and *p53* mutation occurs in various tumors including glioma. The recently updated data from cBioProtal (till December 15, 2016) for Cancer Genomics shows that 39.7% *p53* gene mutation exist in 812 merged cohort of LGG tissues and GBM (TCGA, Cell, 2016), the 90.2% mutation of *p53* in 61 LGG samples (UCSF, Science, 2014), and 20.3% in GBM (TCGA, Cell, 2013), which may suggest a negative association with the pejorative WHO grades of glioma. This is consistent with the total N-cadherin contents in various glioma surgical specimens. However, for different glial cell lines *in vitro*, both U87 and U251 cells express abundant proN-cadherin on the cell surface, although U251 is *p53* mutant glioma cell line, HA, U343, and U87 are all *p53* wild-type [27].

Classical cadherin plays important roles in tumor cell progression [28–30]. Due to the structural difference between proN-cadherin and N-cadherin coupled with the fact that proN-cadherin lacks specific structures mediating cell adhesiveness [21], it has been considered as a nonfunctional precursor of mature N-cadherin for a long time. In 2010, proN-cadherin was first localized in the cell membrane [15]. Since, our western blot analyses confirmed abundant expression of proN-cadherin in the membranes of most gliomas, and among 5 related cell lines, malignant astroglioma cells and glioblastoma stem-like cell derived from U251 have higher expression of proN-cadherin. We believe that the difficulty in explaining the increased mobility of glioma cells was because investigators failed to understand that the “N-cadherin” highly expressed in glioma cell membrane was actually proN-cadherin. We hypothesize that the migration and invasion of malignant glioma cells are mainly due to the abnormally high expression of non-adherent proN-cadherin on the cell surface.

GDNF is approximately five times highly expressed in human malignant gliomas compared to normal human brain tissues [2–3]. Our previous research also indicated that GDNF can interact with N-cadherin [10]. To verify whether GDNF interacts with proN-cadherin, we carried out molecular docking, Co-IP, and IF analyses. Molecular docking graphs of the interaction between GDNF and proN-cadherin showed that GDNF interacts with five AA residues in the EC3 region of proN-cadherins. Simulation experiments also found that GDNF interacts with five pairs of AA residues in the EC3 and EC4 regions of N-cadherin (*i.e*., Arg168-Ser585, Asp172-Asn393, Gln111-Asp450, Arg109-Pro523, and Arg165-Gln433) (data not shown). Another study showed that 69 AA residues in the EC4 region of N-cadherin were necessary and sufficient for EMT [31]. These findings indicated that the results of molecular docking between GDNF and proN-cadherin were credible to a certain degree, and our study did not disprove a possible interaction between GDNF and N-cadherin. Our co-immunoprecipitation and immunofluorescence results further confirmed that GDNF and proN-cadherin could interact with each other.

The wound healing and transwell assays in this study showed that proN-cadherin quantity on the U251 cell membrane positively correlated with cell migration and invasion respectively, whereas there was no obvious correlation as regards mature N-cadherin content. This shows that proN-cadherin mediated GDNF-induced glioma cell invasion and migration. Tumor cells are activated by adherence between surface receptors and certain ECM components, which then secrete a variety of matrix-degrading proteins to degrade the ECM [32] and migrate toward the proteolytic degradation region of the matrix via extending pseudopodia [33–34]. In this study, proN-cadherin altered the adhesion properties of U251 glioma cells, and it mediated GDNF-induced cell motion, presumably because it enhanced heterophilic adhesion between the ECM and U251 glioma cells and accelerated cell activation. We also detected increased MMP-9 secretion by glioma cells.

The core cadherin-catenin complex is composed of cadherin itself, β-catenin, and a-catenin. The integrity of this complex is vital for cell migration during development and metastasis. Seidah's lab reported that higher or lower expressions of proprotein convertases (furin and PC5A) alter proN-cadherin levels on the cell surface [35]. This study affirmed that high cell-surface expression of proN-cadherin accelerate tumor cell invasion and migration. Considering that the extracellular parts of proN-cadherin molecule can't interact with each other as N-cadherin molecules do, we hypothesize that proN-cadherin mediation of GDNF to promote cell invasion and migration might be because GDNF cause homotypic adhesion loss within neighboring cells. To deeply explore how the interaction between GDNF and proN-cadherin influence glioma cell's motility, downstream signaling targets were primarily analyzed using western blot. Our results showed that GDNF significantly changed Tyr860 phosphorylation in the intracellular domain of proN-cadherin, but it did not affect the binding capacities of β-catenin, which might interact with the intracellular domain of proN-cadherin. The cadherin-catenin complex is also regulated by tyrosine phosphorylation and dephosphorylation of β-catenin [36]. According to our western blot result, the phosphorylation level of β-catenin Tyr142 was stable, which suggests the association between β-catenin and a-catenin isn't affected. However, three sites in β-catenin (i.e., Tyr489, Tyr654, and Ser33/37/Thr41) obviously decreased phosphorylation levels when malignant astroglioma cell was stimulated by high GDNF which might suggest a positive feedback effect on the activity of cadherin/catenin core complex.

In conclusion, we affirmed that abundant expression of proN-cadherin exist in the cytomembrane of most gliomas, we demonstrated that proN-cadherin serves as an effective transmembrane signaling molecule that mediates the intracellular signal transmission by neurotrophic factors such as GDNF during glioma cells’ migration and invasion, and we hypothesize that proN-cadherin might cause homotypic adhesion loss within neighboring cells and promote heterotypic adhesion within the ECM through a certain mechanism. Since proN-cadherin has its individual functions different from that of mature N-cadherin, it is worthy to further explore their respective roles and mechanisms involved in glioma occurrence and development.

## MATERIALS AND METHODS

### Tissue materials and ethics statement

The materials are similar to those in our previous study [3]. Six samples of human brain tissue samples from patients with acute brain injury who underwent resection to reduce intracranial pressure and 12 glioma tissue samples (World Health Organization [WHO] Grades I-IV) were collected. All samples were from the Affiliated Hospital of Suzhou University and the affiliated hospital of Xuzhou Medical University. The ethics committee approved the experiments.

### Antibodies and reagents

Antibodies used are as follow; N-cadherin antibody (ab76011, Abcam, Cambridge, UK), proN-cadherin antibody (GTX101141, GeneTex, San Antonio, TX, USA), GDNF antibody (ab18956, Abcam, Cambridge, MA, USA), N-cadherin (phospho Y860) (ab119752, Abcam), p120-catenin (ab92514, Abcam), β-catenin (GTX22982, Genetex, San Antonio, TX, USA), β-catenin phosphorylated antibodies including phospho Y654 (ab24925, Abcam), phospho Y489 (ab138378, Abcam), phospho Y142 (ab27798, Abcam), Ser33/37/Thr41 (9561, CST, Danvers, MA, USA), nestin (ab22035, Abcam, Cambridge, MA, USA), rabbit anti-CD133 (ab19898, proteintech, America), and anti-Caveolin-1 (SAB4200216, Sigma, Aldrich, USA).

### Cell culture and transfection

Human astrocytes-hippocampal cells (HA-h, Sciencell Research Laboratories, San Diego, CA, Cat. No. 1830) were cultured in Astrocyte Medium (Sciencell Research Laboratories, San Diego, CA, Cat. No. 1801), and we only utilized cells which have been subcultured from 3 to 8 generations. The human glioma cell lines U343 and U87 were purchased from the ATCC and U251 from Shanghai Institute of Biological Science. These three types of glioma cell lines were cultured separately in DMEM medium supplemented with 10% fetal bovine serum (FBS) and penicillin-streptomycin at 37°C in a humidified 5% CO_2_ atmosphere. Neurospheres were derived from the U251 cell line cultured in DMEM medium without FBS, supplemented with 10 ng/ml epidermal growth factor (EGF), 10 ng/ml basic fibroblast growth factor (bFGF), and 20 ng/ml B27 supplement (PeproTech, Rocky Hill, NJ, http://www.peprotech.com). Neurosphere cultures were maintained for 5 days before each passage. Western blots and immunofluorescence assays were performed to confirm the stem cells are nestin and CD133+ (data not shown).

The expression vector EF1A-proCDH2-IRES-EGFP was constructed based on the *pro-CDH2* sequence (National Center for Biotechnology Information (NCBI) reference sequence: BC036470.1); we also constructed an EF1A-CDH2-IRES-EGFP vector since the first 477-nt sequence was the precursor region of *pro-CDH2*. The siRNA sequences and the control RNAi sequence based on the 477 bp sequence (pro domain) on the N-terminal of proN-cadherin mRNA (RefSeq, BC036470.1). For the sequences of the control RNA: sense (5′-3′): UUCUCCGAACGUGUCACGUTT, antisense (5′-3′): ACGUGACACGUUCGGAGAATT; and for the sequences of the siRNA RNA: sense (5′-3′) GUGCAGUCUUAUCGAAGGATT, antisense (5′-3′) UCCUUCGAUAAGACUGCACTT. The plasmids and control were transfected into serum-starved cells for 24 h using Lipofectamine 2000 (Invitrogen), continuously cultured and used for experiments during the exponential growth period. Quantitative polymerase chain reaction (Q-PCR) results and western blot analysis results showed that proN-cadherin over-expression and silencing were successful (Supplementary Figure 1). Although transfection with the proN-cadherin siRNA plasmid inevitably decreased the level of mature N-cadherin in the cell membrane, our data showed that the decrease in proN-cadherin was far more significant than that of N-cadherin (data not shown). All experiments were carried out independently at least in triplicates.

### Wound healing assay

U251 cells were seeded in six-well plates and cultured until confluence, making a wound manually by scraping the cell monolayer with a 20-μl pipette tip. After rinsing with PBS, monolayers were photographed (×200) at specific time points to monitor cell migration across the wound. The distance was measured using Adobe PhotoShop CS3 (Adobe, San Jose, CA, USA).

### Transwell invasion experiment

Transwell permeable plates with 8-μm pores (Corning Incorporated, Corning, NY, USA) placed in a 24-well dish coated with Matrigel (BD Biosciences, Franklin Lakes, NJ, USA) were used for the cell invasion assays. Cells were seeded into the top well, and after 24 h the bottom well was fixed and stained with hematoxylin. The invading cells were photographed (×200) at six randomly selected fields and counted.

### Simulation experiment

The homology model of a GDNF dimer based on the crystal structure of *Rattus norvegicus* GNDF-GFRα1 (PDB ID: 3fub, Chain A and D, x-ray diffraction) was constructed using the HOMCOS (http://homcos.pdbj.org/). Because the sequences of human and *Rattus norvegicus* GDNF mRNA (NM_000514.4 and NC_005101.4, respectively) are 100% consistent, the homolog crystal structure of GDNF is extremely credible. The human N-cadherin homolog crystal structure was constructed with Modeler 9v8, and the subsequent protein structure was preserved by energy minimization process. Since there is no N-cadherin crystal template, we chose the crystal structure of *Mus musculus* C-cadherin in cryo-electron tomography as the template protein (PDB ID: 1Q5C, Chain D). The consistency between human N-cadherin mRNA and *Mus musculus* C-cadherin mRNA sequence is 44%. The crystal structure of the pro region of proN-cadherin was directly obtained from the PDB database (ID: 1OP4, nuclear magnetic resonance structure). Docking study of GDNF and proN-cadherin was carried out using hex8.0.0 to explore the potential binding sites and binding energy [37].

### Co-immunoprecipitation and western blot

Cell membrane proteins were extracted using eukaryotic membrane protein extraction kits from tissues or cell lines (Thermo Fisher Corp., Waltham, MA, USA). Briefly, 2 μg of the first antibody was added to the lysates followed by protein A+G agarose (sc-2003, Santa Cruz Biotechnology, Santa Cruz, CA, USA). The beads were washed three times with lysis buffer and boiled in SDS loading buffer to elute the precipitated proteins.

Samples were separated on 10% SDS-PAGE gels. After transfer to the polyvinylidene fluoride (PVDF) membranes, blots were blocked with 3% BSA and corresponding primary and secondary antibodies diluted in TBS-Tween 20 was added, then scanned using an Odyssey imaging system (LI-COR Biosciences, Lincoln, NE, USA).

### Immunofluorescence

Cells were cultured on coverslips for 24 h. After washing with PBS, cells were fixed for 30 min at room temperature in 4% formaldehyde. Fixed cells were permeabilized for 5 min using 0.3% Triton X-100, blocked for 30 min using 5% goat serum/PBS, and then immunostained using primary antibodies overnight at 4°C. After washing with PBS, the secondary antibody (EarthOx Life Sciences, Millbrae, CA, USA) was applied for 1 h at room temperature in the dark. Nuclei were stained with DAPI and fluorescence images were captured with a fluorescent inverted microscope (Leica Microsystems, Wetzlar, Germany).

### Adhesion experiment

Ninety-six-well plates were pre-coated with 100 μg/ml collagen I (human collagen Type I, cat. no.: 2227128, Millipore Corporation, Billerica, MA, USA), IV (Collagen IV full-length protein, ab7536, Abcam), human fibronectin (human plasma, cat. no.: 2326977, Millipore Corporation) or laminin (human fibroblasts, L4544, Sigma-Aldrich, St. Louis, MO, USA) at 4°C overnight. Then, cells were plated at 50,000/well. After incubation at 37°C for 2 h, nonadherent cells were removed by washing with PBS. Adherent cells were fixed with 4% paraformaldehyde, stained with 0.5% crystal violet for 2 h.; cells were washed using distilled water and lysed in 2% SDS solution. Adhesion was quantified by measuring absorbance at 570 nm using a Bio-Tek Synergy 2 plate reader.

### Enzyme-linked immunosorbent assay (ELISA)

Samples in each group were collected in sterile tubes and centrifuged at 1000 rpm for 15 min to obtain supernatants. Supernatants were determined according to the manufacturer's instructions (EK0465, Boster, Pleasanton, CA, USA) at least in triplicate dilution series. Results are presented as picograms of MMP-9 per ml.

### Statistical analyses

All analysis was performed using replicates of n>=3. Error bars represent standard errors of the mean. Data were analyzed with Student's *t*-tests and one-way ANOVA. The differences were considered statistically significant at P<0.05.

## SUPPLEMENTARY MATERIALS FIGURES AND TABLES


