# Supplementary material for: Precursor N-cadherin mediates glial cell line-derived neurotrophic factor-promoted human malignant glioma
Source: Oncotarget. 2017 Feb 12;8(15):24902–14. doi: 10.18632/oncotarget.15302 (PMC5421898; doi:10.18632/oncotarget.15302)
Supplement: Supplementary file 1 [file oncotarget-08-24902-s001.pdf]

Precursor N-cadherin mediates glial cell line-derived neurotrophic factor-promoted human malignant glioma

SUPPLEMENTARY FIGURE AND TABLE

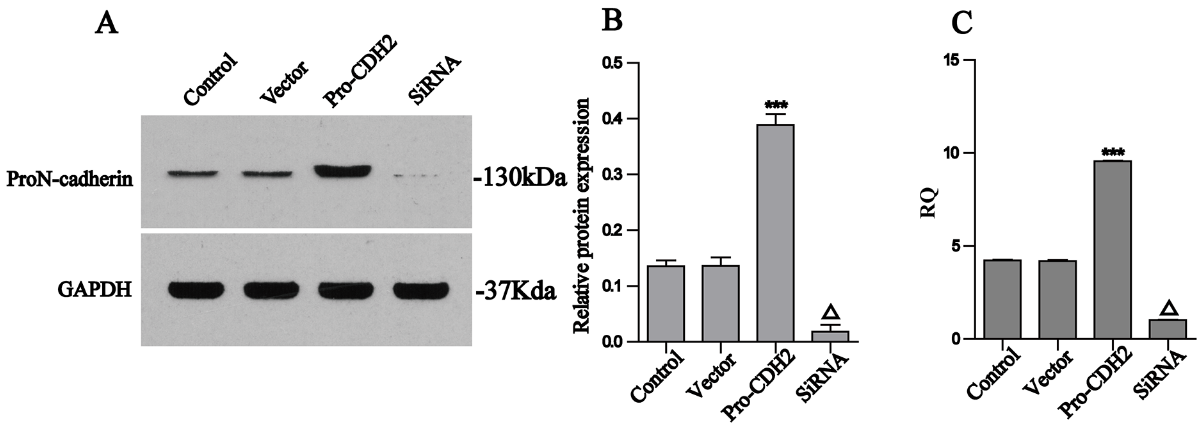

Supplementary Figure 1: Western blotting and quantitative RT-PCR analysis of proN-cadherin expression level with pro-CDH2 and siRNA treatment. A and **B.** Western blotting analysis of proN-cadherin expression level in U251 cells transfected with pro-CDH2 and siRNA, respectively. GAPDH served as a loading control. B. The bar graphs show the relative expression of the proN-cadherin protein among the groups. **C.** RT-PCR shows the transcriptional levels of the proN-cadherin gene with vector used as a loading control. ***P<0.001, compared with the vector groups.

Supplementary Table 1: Human Brain tissue samples

	Glioma tissue												Normal brain tissue					
**Number**	T1	T2	T3	T4	T5	T6	T7	T8	T9	T10	T11	T12	N1	N2	N3	N4	N5	N6
**Age**	29	33	48	38	19	45	49	15	48	37	38	15	45	38	45	44	61	42
**Sex**	M	F	M	M	M	M	M	M	M	M	F	F	M	M	M	M	M	M
